# Supplementary material for: Bi-Directional Interactions between Glucose-Lowering Medications and Gut Microbiome in Patients with Type 2 Diabetes Mellitus: A Systematic Review
Source: Genes (Basel). 2023 Aug 1;14(8):1572. doi: 10.3390/genes14081572 (PMC10454120; doi:10.3390/genes14081572)
Supplement: Supplementary file 1 [file genes-14-01572-s001.zip › Supplementary Material S1.pdf]

Supplementary material S1

Executed search strings for each database

Embase

('intestine flora'/exp OR Microflora/de OR microbiome/de OR 'bacterial microbiome'/de OR 'host microbe interaction'/de OR (((intestin* OR gut OR colon* OR gastrointestin*) NEAR/3 (flora OR microflora OR microb* OR bacteria*)) OR microbiome* OR microflora*):ab,ti) AND ('drug interaction'/exp OR 'drug response'/de OR 'antidiabetic activity'/exp OR 'antidiabetic agent'/ exp OR 'pharmacokinetics'/exp OR 'pharmacokinetics':lnk OR glycosidase/exp OR 'glycosidase inhibitor'/exp OR (drug* OR antibiot* OR antidiabet* OR anti-diabet* OR metformin* OR acarbose OR ((glucagon OR dipeptide*) NEAR/3 peptid* NEAR/3 (agonist* OR inhibitor*)) OR thiazolidin* OR gliptin* OR (sodium NEAR/3 glucose NEAR/3 inhibitor*) OR glifozin* OR sulfonylurease* OR pharmacokinetic* OR (drug NEAR/3 (absorpt* OR action* OR interaction* OR accumulat* OR activat* OR adsorpt* OR bioavailabilit* OR clearan* OR dialysabilit* OR diffuse* OR disposit* OR distribut* OR eliminat* OR excret* OR half-life OR inactivat* OR metabol* OR penetrat* OR release* OR retention*)) OR glucosidase* OR glycosidase* OR hypoglycem* OR hypoglycaem*):ab,ti) AND ('diabetes mellitus'/de OR 'non-insulin dependent diabetes mellitus'/de OR 'antidiabetic activity'/de OR 'antidiabetic agent'/de OR 'diabetic patient'/de OR (diabet* OR antidiabet* OR T2D OR T2DM OR DMII OR DM2 OR NIDDM):ab,ti) NOT ([animals]/lim NOT [humans]/lim) NOT ((type-1 OR type-i) NOT (type-2 OR type-ii)):ab,ti NOT ([Conference Abstract]/lim) NOT ([letter]/lim)

PubMed

(Gastrointestinal Microbiome / OR Microbiota / OR Host Microbial Interactions / OR (((intestin* OR gut OR colon* OR gastrointestin*) ADJ3 (flora OR microflora OR microb* OR bacteria*)) OR microbiome* OR microflora*).ab,ti.) AND (exp Drug Interactions/ OR exp Hypoglycemic Agents/ OR exp Pharmacokinetics/ OR pharmacokinetics.fx. OR exp Glycoside Hydrolases/ OR (drug* OR antidiabet* OR anti-diabet* OR metformin* OR acarbose OR ((glucagon OR dipeptide*) ADJ3 peptid* ADJ3 (agonist* OR inhibitor*)) OR thiazolidin* OR gliptin* OR (sodium ADJ3 glucose ADJ3 inhibitor*) OR glifozin* OR sulfonylurease* OR pharmacokinetic* OR (drug ADJ3 (absorpt* OR action* OR interaction* OR accumulat* OR activat* OR adsorpt* OR bioavailabilit* OR clearan* OR dialysabilit* OR diffuse* OR disposit* OR distribut* OR eliminat* OR excret* OR half-life OR inactivat* OR metabol* OR penetrat* OR release* OR retention*)) OR glucosidase* OR glycosidase* OR hypoglycem* OR hypoglycaem*).ab,ti.) AND (Diabetes Mellitus/ OR "Diabetes Mellitus, Type 2"/ OR Hypoglycemic Agents/ OR (diabet* OR antidiabet* OR T2D OR T2DM OR DMII OR DM2 OR NIDDM).ab,ti.) NOT (exp animals/ NOT humans/) NOT ((type-1 OR type-i) NOT (type-2 OR type-ii)).ab,ti. NOT (news OR congres* OR abstract* OR book* OR chapter* OR dissertation abstract* OR letter*).pt.

Web of Science Core Collection

TS=(((((intestin* OR gut OR colon* OR gastrointestin*) NEAR/2 (flora OR microflora OR microb* OR bacteria*)) OR microbiome* OR microflora*)) AND ((drug* OR anti-diabet*) NEAR/2 (metformin* OR

acarbose OR ((glucagon OR dipeptide*) NEAR/2 peptid* NEAR/2 (agonist* OR inhibitor*)) OR thiazolidin* OR gliptin* OR (sodium NEAR/2 glucose NEAR/2 inhibitor*) OR glifozin* OR sulfonylurease* OR pharmacokinetic* OR (drug NEAR/2 (absorpt* OR action* OR interaction* OR accumulat* OR activat* OR adsorpt* OR bioavailabilit* OR clearan* OR dialysabilit* OR diffuse* OR disposit* OR distribut* OR eliminat* OR excret* OR half-life OR inactivat* OR metabol* OR penetrat* OR release* OR retention*)) OR glucosidase* OR glycosidase* OR hypoglycem* OR hypoglycaem*)) AND ((diabet* OR antidiabet* OR T2D OR T2DM OR DMII OR DM2 OR NIDDM)) NOT ((animal* OR rat OR rats OR mouse OR mice OR murine OR dog OR dogs OR canine OR cat OR cats OR feline OR rabbit OR cow OR cows OR bovine OR rodent* OR sheep OR ovine OR pig OR swine OR porcine OR veterinar* OR chick* OR zebrafish* OR baboon* OR nonhuman* OR primate* OR cattle* OR goose OR geese OR duck OR macaque* OR avian* OR bird* OR fish*) NOT (human* OR patient* OR women OR woman OR men OR man)) NOT ((type-1 OR type-i) NOT (type-2 OR type-ii))) AND DT=(article)

Google Scholar

flora|microflora|microbioma|bacteria drug| antidiabetics|" |drug interaction|metabolism" diabetes
